# 884 Factors Influencing Emergency Department Length of Stay in Pediatric Burn Patients

**DOI:** 10.1093/jbcr/iraf019.415

**Published:** 2025-04-01

**Authors:** Nishant Kumar, Katherine Oag, Lisa Vitale, Mariah Malaniak, Justin Klein, Elika Ridelman, Christina Shanti

**Affiliations:** Children’s Hospital of Michigan, Wayne State University School of Medicine; Children’s Hospital of Michigan, Wayne State University School of Medicine; Children’s Hospital of Michigan, Wayne State University School of Medicine; Children’s Hospital of Michigan, Wayne State University School of Medicine; Children’s Hospital of Michigan, Wayne State University School of Medicine; Children’s Hospital of Michigan, Wayne State University School of Medicine; Children’s Hospital of Michigan, Wayne State University School of Medicine

## Abstract

**Introduction:**

Globally, the demand for pediatric emergency department (ED) visits has increased, leading to extended lengths of stay (LOS) in the ED. Various factors such as time of arrival, triage category, pain duration, imaging, medications, and reduced inpatient bed availability contribute to this trend. This study aims to identify the factors affecting ED length of stay in pediatric patients presenting with burn injuries.

**Methods:**

A retrospective chart review was conducted on patients under 18 years of age who presented to an American Burn Association (ABA)-verified urban pediatric burn center between March 1, 2023, and March 1, 2024, with burn injuries and an ED stay of four or more hours. Data on patient demographics, clinical presentation, treatment, and ED LOS were collected.

**Results:**

The study included 300 pediatric patients (159 males, 141 females), with an average ED length of stay of 6 hours and 12 minutes (372 minutes). Patients arriving from a burn center outpatient office or clinic (x ®=471 min, SD=249 min) had significantly longer ED stays compared to those arriving directly from the site of injury (x ®=364 min, SD=94 min) (p=0.024) or via transfer from another ED (x ®=362 min, SD=111 min) (p=0.019). Additionally, patients with an ED disposition of outpatient-in-bed (OPIB) (x ®=438 min, SD= 192 min) had significantly longer ED stays than those discharged directly home (x ®=362 min, SD=101 min) (p=0.013). Extended ED LOS was positively correlated with longer times between triage and placement in a treatment room (x ®=53 min, 95% CI [47 min,60 min], p< 0.001), IV initiation and burn wound debridement (x ®=105 min, 95% CI [93 min,118 min], p< 0.001), notification and arrival of the burn resource nurse (x ®=63 min, 95% CI [51 min,75 min], p< 0.001), and the duration between the nurse’s arrival and completion of burn debridement (x ®=100 min, 95% CI [88 min,111 min], p=0.048). Interestingly, there were no significant differences in ED LOS for patients based on mechanisms of injury (p=0.179) or percent total burn surface area (p=0.306).

**Conclusions:**

Extended ED length of stay for pediatric patients presenting with burn injuries appears to be linked to small burns that require overnight stays (OPIB disposition) to ensure appropriate consulting services and wound care teaching is performed. Other factors that contribute to an increase in ED LOS are delays in triage, IV start time, and burn resource team response.

**Applicability of Research to Practice:**

Identifying factors that affect length of stay in the ED for burn patients can help providers anticipate the patient’s expected length of stay and avoid unnecessary time spent in the ED. This information gives providers the ability to effect change to the process and deliver more effective and efficient treatments, thus providing more cost-effective care and improve patient outcomes.

**Funding for the Study:**

Foundation Funding

